# Olfaction and dementia: A review of the current state of research

**DOI:** 10.1002/alz70858_103018

**Published:** 2025-12-26

**Authors:** Federica D'Andrea

**Affiliations:** ^1^ The Geller Institute of Ageing and Memory, University of West London, London, London, United Kingdom

## Abstract

**Background:**

There is a growing interest in using olfactory (smell) stimulation in dementia care. The sense of smell plays an important role in everyday life. It enables humans to detect and differentiate between various scents, mediates the flavours of foods and drinks, and serves as a first warning signal, detecting smoke from a fire, leaking natural gas or spoiled food. There is also evidence demonstrating the power of smells in triggering autobiographical memories and emotions, given the close relationship between olfaction, memory, and emotional processes at the neuroanatomical level. Despite its increasing in popularity in clinical practice, limited studies evaluate the effectiveness of olfactory interventions.

**Aims:**

This work aims to i) synthesis the current state of research on dementia and olfaction interventions; ii) and identify current evidence on odour‐evoked memory by people living with dementia.

**Methods:**

Two rapid reviews are used, including an update published rapid review on the efficacy of interventions using olfactory stimulation in dementia care; and an ongoing mixed‐method rapid review on odour‐evoked memories for people living with dementia. The search is conducted across five databases (Medline, Academic Search Elite, PsycARTICLES, CINAHL, PsycINFO). Eligible studies are assessed for methodological quality and synthesised using a narrative approach.

**Results:**

Findings suggest mixed results on the benefits of olfactory stimulation on responsive behaviours and cognitive function. Although the evidence available is limited, encouraging results were found regarding olfactory stimulation and increased autobiographic memories, sleep duration, food intake and improved balance.

**Conclusion:**

Olfactory intervention in dementia care is an emergent area of research warranting attention. This work discussed the important role of smell and the current state‐of‐the‐art in dementia and olfaction research, and its contribute to overall well‐being of people with dementia.

